# Quantitative analysis of intra- and inter-individual variability of human beta-cell mass

**DOI:** 10.1038/s41598-017-16300-w

**Published:** 2017-11-27

**Authors:** Scott K. Olehnik, Jonas L. Fowler, Gil Avramovich, Manami Hara

**Affiliations:** Department of Medicine, The University of Chicago, Chicago, Illinois 60637 USA

## Abstract

Pancreatic beta-cell mass is a critical determinant of the progression of diabetes. The loss of beta-cells in various types of diabetes has been documented in comparison to age, sex and body mass index (BMI) matched control subjects. However, the underlying heterogeneity of beta-cell mass in healthy individuals has not been considered. In this study, the inter-individual heterogeneity in beta-cell/islet mass was examined among 10 cases of age-matched non-diabetic male subjects in relation to BMI, pancreas weight, and the percent ratio, volume and number of islets in the whole pancreas. Beta-cell/islet mass was measured using a large-scale unbiased quantification method. In contrast to previous studies, we found no clinically relevant correlation between beta-cell/islet mass and age, BMI or pancreas weight, with large differences in beta-cell/islet mass and islet number among the individuals. Our method extracts the comprehensive information out of individual pancreas providing multifaceted parameters to study the intrinsic heterogeneity of the human pancreas.

## Introduction

In the human pancreas, there is clear intra-individual regional variability in beta-cell/islet mass, with marked fluctuation within each region and throughout the pancreas^1,2^, which is likely to lead to sampling bias, when specific tissue blocks are examined. In addition, selection of “islet-containing areas” out of the entire section results in overestimation. We have shown a stereological quantification method of beta cell/islet mass in the whole human pancreas^3^. Based on our simulation, such biased selection could result in 4.5 to 8-fold overestimation compared to the entire section analysis. Point counting morphometry could increase the range of overestimation, 4 to 14-fold. In past studies, the accuracy may have been hampered due to methodology and sampling bias^4–14^.

In comparative studies, control subjects are selected as age-, sex- and BMI-matched, which is largely based on the article from Saisho *et al*. with conclusion that beta-cell mass increases with BMI^13^, although the authors noted “considerable variance in beta-cell mass not explained by BMI (r = 0.5)”. Rahier *et al*. also reported notable overlap of beta-cell mass between the lean and the obese subjects, where their conclusion was that “Obesity has a modest impact on beta-cell mass in humans (r = 0.3), when compared with rodents”^11^. Interestingly, while a study conducted in Korea showed positive correlation^10^, another in Japan reported a negative correlation^14^. Although the number of donors was relatively large, notably limited areas out of the whole pancreas were examined in all studies. Sampling was restricted to 1 block each per body and tail^11^, 1 block per body-tail (dorsal)^10^ or tail only^13^. Considering the intra-individual variability of beta-cell mass, additional overestimation of beta-cell mass is likely anticipated. Reference for pancreas volume to estimate the absolute mass of beta-cells is critical, where pancreas weight is the most appropriate. Saisho *et al*. estimated pancreas volume based on computed tomography scanned images of live humans and used the population-based volume data^13^. Kou *et al*. utilized the same method^14^.

We reason that it is essential to fully understand the basic facts about the human pancreas using unbiased quantification methods, and importantly in the whole pancreas. Here we show a method to quantitatively analyze the whole human pancreas, which includes the assessment of islet number in individuals.

## Results

### Whole pancreas analysis

**Figure 1:**
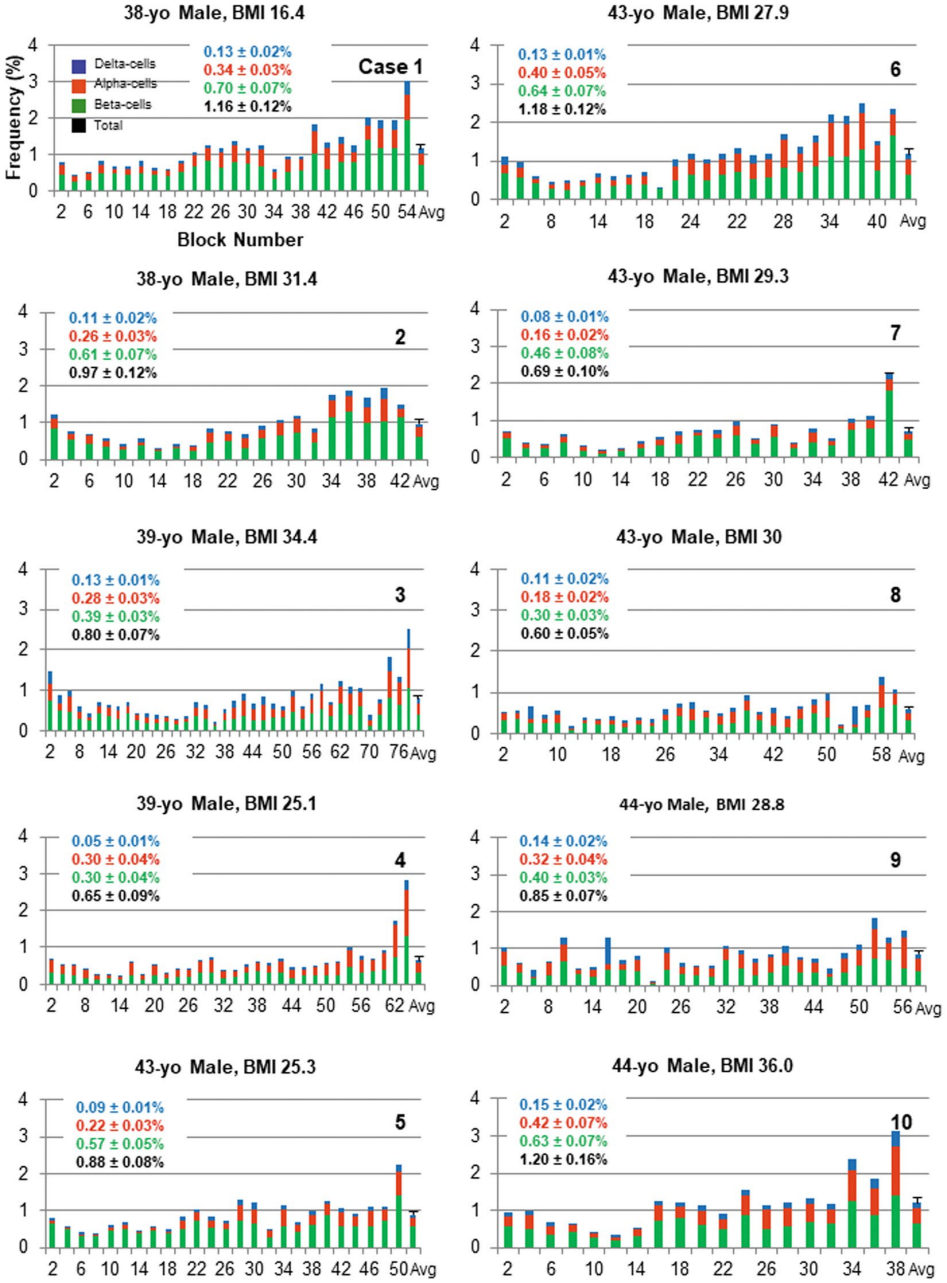
Whole pancreas analysis. Regional distribution of endocrine cells throughout the whole pancreas, beta-cells in green, alpha-cells in red and delta-cells in blue. Ten cases of age-matched non-diabetic male subjects are shown. X-axis: Block number from head, body to tail region (from left to right). All the graphs are plotted in the same scale for a better comparison. Each pancreas was divided into consecutive tissue blocks, with preparation alternating between fresh-frozen and paraffin embedding. The latter set of tissue blocks was used in the present study. The last bar in each plot depicts average ± SEM, which value is shown in the inset.

Distribution of pancreatic endocrine-cells was examined throughout the whole pancreas of 10 cases of age-matched non-diabetic male subjects (41.4 ± 2.6 year old), using a large-scale unbiased quantification method^3^. Figure 1 depicts remarkable intra-individual variability as well as inter-individual heterogeneity in beta-cell/islet mass. Overall, regional distribution of beta-cell/islet mass shows a gradual increase from head to tail region, although it fluctuates to a great extent.

### Heterogeneity of the human beta-cell/islet mass

**Figure 2:**
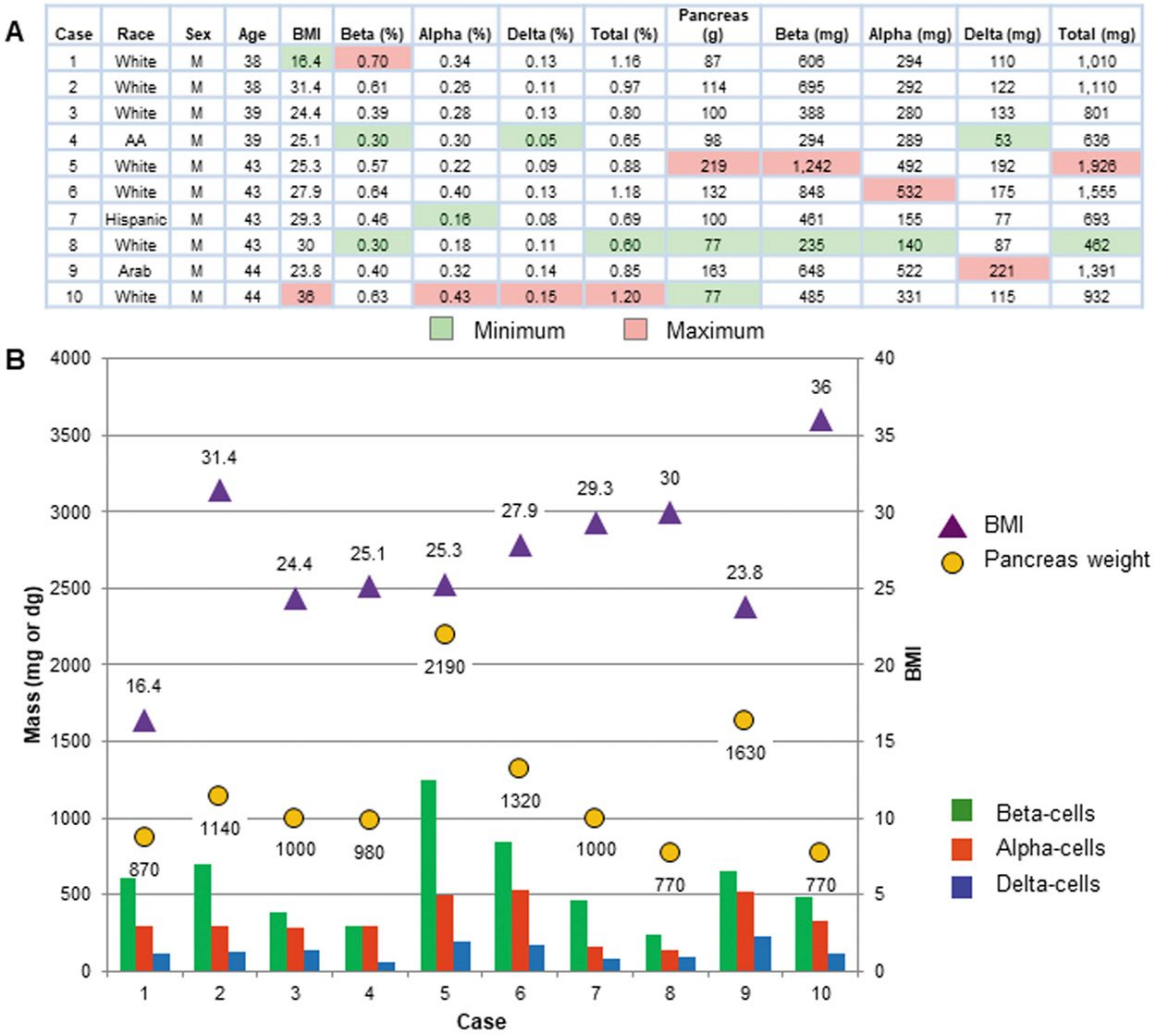
Heterogeneity of the human beta-cell/islet mass. (**A**) The ten cases examined for relationship between beta-cell/islet mass and BMI, pancreas weight, and the percent ratio and the volume of beta-, alpha- and delta-cells. The minimum and maximum values in each column are highlighted in light green and orange, respectively. (**B**) Summary of the results is shown as a compiled graph.

Next, the inter-individual heterogeneity in beta-cell/islet mass was examined in the 10 cases (Fig. 2). Figure 2A includes the results of the whole pancreas analysis shown in Fig. 1. The highest ratio of beta-cell mass was observed in Case 1 (0.7%), who was a lean subject with BMI 16.4. In contrast, the lowest beta-cell ratio of 0.3% was recorded by Case 4, an overweight subject (BMI 25.1) and Case 8, an obese subject (BMI 30.0). Case 10 whose BMI was highest (36.0) had the smallest pancreas (77 g) similarly to Case 8. In fact, Case 8 exhibited a number of the minimum values, which further included the volume of beta- and alpha-cells and the total endocrine-cells (235, 140 and 462 mg, respectively). Case 5 stands out with his large pancreas (219 g), resulting in the highest endocrine-cell mass (~2 g), which is 2–4 fold larger than the rest. One particular difference in Case 5 was his height of 195 cm. In any comparative studies, such a subject should be considered as “an outlier” and not be appropriate as “a control”. In Case 4, the ratio of beta-cells was the same as that of alpha-cells (0.3%), whereas the rest of the cases showed the well-known consistent gradient of “beta-cell mass > alpha-cell mass”. These results are plotted in Fig. 2B for a better comparison.

### Islet size distribution and cellular composition

**Figure 3:**
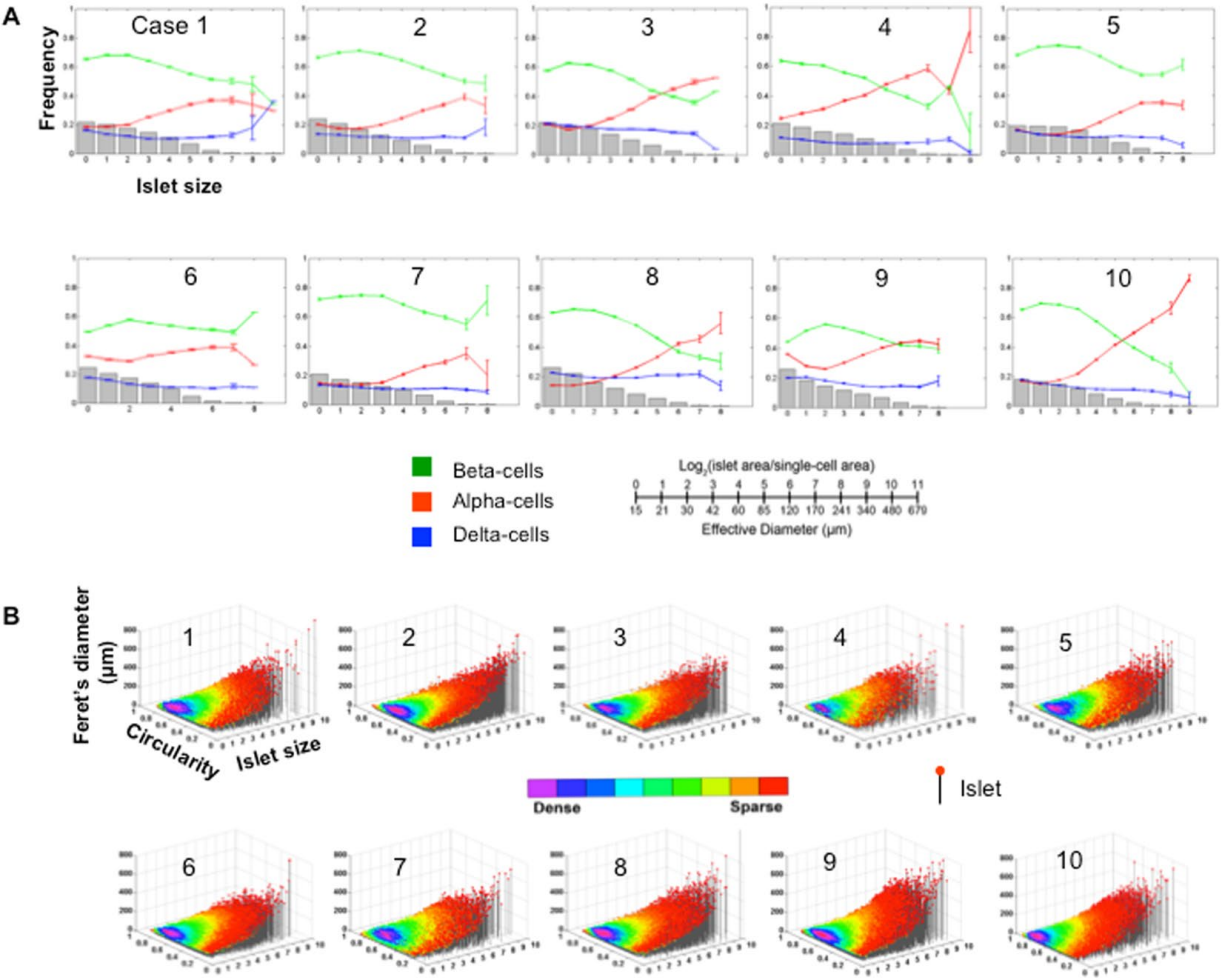
Islet size distribution, cellular composition. (**A**) Quantitative analysis of individual islet size distribution and cellular composition. Relative frequency of islet size (gray bar) and ratios of beta (green), alpha (red), and delta (blue) cells within islets are plotted against islet size; means ± SEM. Note that islet size is presented as a logarithmic scale considering the high number of small islets and the low number of large islets. In addition, islet area is divided by the single-cell area (178 μm^2^)^20^ to make them as dimensionless values representing the number of cells in a given islet area. See the conversion between logarithmic islet area (logarithmic) and effective diameter (μm). (**B**) 3D visualization of islet size and shape distribution. Each dot represents a single islet/cluster with reference to size (area) and shape. Circularity reports the degree of roundness of a structure, where 1.0 corresponds to a perfect circle. Feret’s diameter is the longest distance within a structure. Circularity and Feret’s diameter are closely related that together depict a shape of a given structure. The density of islets is color-coded from sparse to dense.

Dynamic changes of cellular composition throughout the islet size distribution were observed in each case (Fig. 3A). Beta-cells compose roughly ~60% of the total endocrine cells in small islets (<100μm in diameter). In Cases 1, 2, 5, 6 and 7, beta-cells were the major population throughout the islet size distribution with an increased alpha-cell ratio in larger islets. The rest showed an inverted ratio of beta- and alpha-cells in larger islets (>100μm in diameter). Interestingly, they all had a beta/alpha ratio of <2.0. Particularly Case 4 with the ratio of 1.0 exhibited the most complex pattern of cellular composition. In contrast, delta-cell ratios appear to be constant in all subjects. Three-dimensional scatter plots visualize islet size (area) and shape (circularity and Feret’s diameter) distribution (Fig. 3B). Large islets are sparse (single islets in red dots) and tend to be elongated in structure.

### Assessment of the number of islets in the ten cases

**Figure 4:**
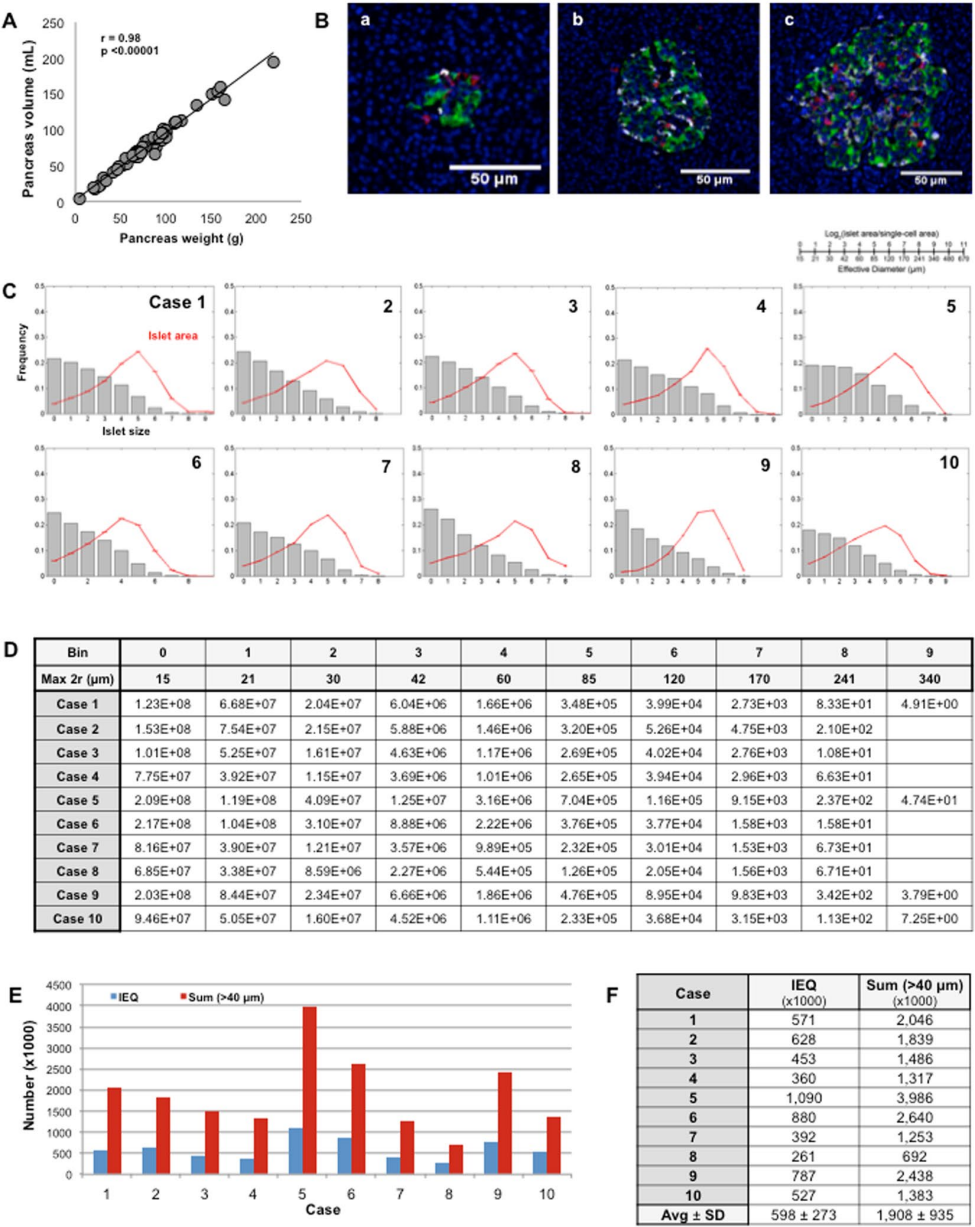
Assessment of the number of islets in the ten cases. (**A**) Pancreas tissue density. (**B**) Representative islets from small to large in size. (**C**) Fraction of islet size distribution (gray bar) and total islet area (red line). (**D**) The estimated number of islets in each bin. (**E**) Estimated number of IEQ (i.e. the unit of standardized islet mass used in clinical islet transplantation to assess the yield of isolated islets from donor pancreata) and islets with a diameter greater than 40 µm in the ten cases. (**F**) Summary of the islet numbers.

The whole pancreas analysis and the measurement of pancreas weight enabled us to assess the number of islets in individuals. It was calculated as follows: Endocrine-cell mass (mg × 10^−3^)/Volume of islets (μm^3^ × 10^12^), based on our accumulated data of pancreas weight and volume, which indicates an approximate density of 1 g/mL (Fig. 4A; n = 64, age 47.5 ± 18.7). Representative islets from small to large in size are shown in Fig. 4B,A–C, which fall into the bins of 3 (30–42 μm in diameter), 5 (60–85 μm) and 7 (120–170 μm), respectively. Fig. 4C depicts the relative contribution of each bin of islets to total endocrine-cell area. The greater number of endocrine-cell clusters and small islets did not markedly share the total area. Overall, the contribution peaked around islets with 60–120 µm in diameter. The estimated number of islets in each bin is summarized in Fig. 4D. Conversion of the number of islets to Islet Equivalent (IEQ:150 µm in diameter) shows a similar range of reported islet yields^15,16^, which is plotted in blue together with the number of islets with a diameter greater than 40 µm in red (Fig. 4E). Figure 4F summarizes these numbers of each donor.

## Discussion

Three major sources of biased measurement of beta-cell/islet mass in the human pancreas are: [1] sampling specific pancreatic region(s) out of the whole pancreas; [2] restricted selection of islet-rich areas out of the entire section; and [3] reference to the population-based volume data, instead of individual pancreas weight. In the present study, we aim to demonstrate the importance of whole pancreas analysis with unbiased quantification methods to precisely assess beta-cell/islet mass. We assessed the number of islets in relation to islet size in individual donor pancreata of various organ weight. Such assessment of the islet number has not been attempted in the past. The total number of islets can be converted to IEQ. Islet donor score used for optimal pancreas/donor selection for clinical islet transplantation includes BMI, where in general it is considered that the higher the better^17^. This study showed otherwise, for example, that Case 1 with BMI 16.4 had similar amount of endocrine cells (1.0 g) with Case 2 with BMI 31.4 (1.1 g). However, in practice, donors with BMI less than 20 are likely to be declined. Successful islet isolation is currently defined as post-purification islet yield over 400,000 (400 K) IEQ, which is considered to have been free from technical deviations or mistakes^15,16^. Interestingly, the estimated IEQ of the three Cases 4, 7 and 8 in the present study was below 400 K with their BMI being 25.1, 29.3 and 30, respectively. This threshold is clinically ideal, which ensures islet transplantation for recipients with up to 80 kg body weight (5 K IEQ per kg). Some donors will not reach that criterion due to inter-individual heterogeneity.

The inter-individual heterogeneity of human beta-cell mass holds important implications to other diabetes research as well. In stem cell research, many investigators are making tremendous efforts to generate surrogate beta-cells from various sources, mainly human embryonic stem cells and induced pluripotent stem cells. Here, the ultimate target population is children and juveniles with diabetes, who are not eligible for islet transplantation, nor (often times) for medications approved for adults. Eligibility criteria for recipients of islet transplantation are adult patients with type 1 diabetes (age 18 to 65 years) either having severe hypoglycemia unawareness or having had a kidney transplant and are taking immunosuppressant drugs (Diabetes Research Wellness Foundation). Generation of functional surrogate beta-cells should benefit all ages of patients. Currently, little is known about how many beta-cells will be necessary to cure diabetes in children and juveniles. Non-invasive *in vivo* imaging of the beta-cell mass is well-desired technology. However, how would we predict people at a risk of developing diabetes with information of the absolute mass of beta-cells alone? This therapeutics may need an additional marker that reports the basal pool of individual beta-cells^18^, such as alpha-cell mass that is relatively stable even under autoimmune destruction of beta-cells^19^.

In summary, there is a lack of rigor in recognizing and further understanding the underlying heterogeneity of beta-cell mass in healthy individuals. This study provides an experimental simulation of how control selection would affect comparative studies of any kind. To examine beta-cell/islet mass of a 40-year old male with diabetes, for example, selecting 10 cases of age-matched non-diabetic males as a control are more than sufficient. However, our results suggest that the underlying heterogeneity in control subjects can confound this selection process. For method development and proof of concept, we only focused on 10 cases of age-matched controls at a single time point in the present study. We are currently developing a tissue bank and online database of human pancreatic images from many more cases with different age ranges. Our goal is to analyze over 100 samples and provide raw data as well as our measurements of beta-cell/islet mass to the scientific community. Ultimately, this data can be used in a variety of ways, but most notably, we would like to develop a model for explaining inter-individual heterogeneity of human beta-cell mass. It is important to establish the basic facts about the beta-cell mass and the number of islets in humans throughout the lifespan, in order to lay the foundation for the development of critical technologies for a cure for diabetes.

## Materials and Methods

### Human pancreas specimens

Human pancreata were generously provided by the Gift of Hope Organ Procurement Organization in Chicago and the nPOD. Written informed consent from a donor or the next of kin was obtained for use of a sample in research. The use of deidentified human tissues in the study was approved by the Institutional Review Board at the University of Chicago.

### Microscope and computing platforms

Microscopic images were taken with an Olympus IX8 DSU spinning disk confocal microscope (Melville, NY) with imaging software StereoInvestigator (MicroBrightField, Williston, VT). Large-scale image capture and computer-assisted semi-automated analysis of the whole tissue section are previously described in detail, including all macros used and a video demonstration^3^. Briefly, image analysis was performed using custom-written scripts for Fiji/ImageJ (http://rsbweb.nih.gov/ij/). Total islet area that includes unstained fractions such as intraislet capillaries and other endocrine cells such as pancreatic polypeptide- and ghrelin-cells, was measured by automated contouring of each islet structure. Circularity and Feret’s diameter depict the shape of islets such that an elongated islet possesses low circularity and a long Feret’s diameter. MATLAB (MathWorks, Natick, MA) was used for mathematical analyses.”

### Immunohistochemistry

The pancreas was divided into consecutive tissue blocks (~5 mm) with alternating collection between paraffin-embedding and snap frozen18. In this study, paraffin-embedded sections (5 μm) were stained with the following primary antibodies (all 1:500): polyclonal guinea pig anti-porcine insulin (DAKO, Carpinteria, CA), mouse monoclonal anti-human glucagon (Sigma-Aldrich, St. Louis, MO), polyclonal goat anti-somatostatin (Santa Cruz, Santa Cruz, CA), and DAPI (Invitrogen, Carlsbad, CA). The primary antibodies were detected using a combination of DyLight 488, 549 and 649-conjugated secondary antibodies (1:200, Jackson ImmunoResearch Laboratory, West Grove, PA).

### Data Availability

All data generated or analyzed during this study are included in this article or are available from the corresponding author.

All methods were performed in accordance with the relevant guidelines and regulations.
